# The Role of Thermodynamic and Informational Entropy in Improving Real Estate Valuation Methods

**DOI:** 10.3390/e25060907

**Published:** 2023-06-07

**Authors:** Ünsal Özdilek

**Affiliations:** Business School, Department of Strategy, Social and Environmental Responsibility, University of Quebec, Montreal, QC H3C 3P8, Canada; ozdilek.unsal@uqam.ca

**Keywords:** value state measure, real estate appraisal, evaluation methods, information, Entropy weighting method (EWM), multi-criteria decision making (MCDM)

## Abstract

Price, Cost and Income (PCI) are distinct economic indicators intrinsically linked to the values they denote. These observables take center stage in the multi-criteria decision-making process that enables economic agents to convey subjective utilities of market-exchanged commodities objectively. The valuation of these commodities heavily relies on PCI-based empirical observables and their supported methodologies. This valuation measure’s accuracy is critical, as it influences subsequent decisions within the market chain. However, measurement errors often arise due to inherent uncertainties in the value state, impacting economic agents’ wealth, particularly when trading significant commodities such as real estate properties. This paper addresses this issue by incorporating entropy measurements into real estate valuation. This mathematical technique adjusts and integrates triadic PCI estimates, improving the final stage of appraisal systems where definitive value decisions are crucial. Employing entropy within the appraisal system can also aid market agents in devising informed production/trading strategies for optimal returns. The results from our practical demonstration indicate promising implications. The entropy’s integration with PCI estimates significantly improved the value measurement’s precision and reduced economic decision-making errors.

## 1. Introduction

The heart of economics, and more specifically, appraisal practice, primarily revolves around interpreting and quantifying the market value of real assets [1,2,3]. In North America, property valuation has been well-organized and improving for more than a century, benefiting from the works of classical economists [4,5,6,7,8,9,10] and contemporary authors proposing the concurrent use of triadic concepts, observables and approaches [11,12,13]. Improvements in value estimations have been achieved using statistical modeling approaches, benefiting from non-linear, automatized, and intelligent algorithms, complex tools and rich data [14,15,16,17]. However, these models are limited by the same conventional framework of appraisal systems, which integrate some improvements but cannot go beyond the boundary of existing concepts, observables and methods.

Indeed, market value estimation for a given property, herein referred to as the “subject”, can be achieved using the three fundamental methods of price, cost and income, each involving detailed computation steps [18]. Considering the triadic responses to the market value of the subject property simultaneously provides the most accurate approximation at this final computational step. However, this requires a careful amalgamation of responses and adjustments of their relevance as the estimates can fluctuate significantly, even for the same property [19,20]. This discrepancy presents a critical gap at the decisive step of the appraisal system, which either relies solely on one method, excluding the results of the other two, or employs subjective weighted averages, posing the risk of bias in the measures and intrusive blending of logical computations from preceding steps [21,22]. To tackle these constraints, and drawing upon the principles of informational and thermodynamic entropies [23], we suggest incorporating the Entropy weighting method (EWM) into the final step of the appraisal system, employing it as a primary value metric alongside traditional PCI methods.

In the last step of the evaluation process, working with the results of the EWM, we remarked that it has the potential to improve the current system of evaluation in a global manner as well as in a case-by-case basis. In addition to this global approach that estimates a fused value for all the responses in the whole data, we also tried the EWM in case-by-case evaluations, adjusting each imperfect estimation of conventional approaches. In that perspective, we improved and adjusted the EWM in several regards, for instance, by integrating the Kullback-Leibler divergence in relative entropies of each case in the data. This is another important contribution of our work as it not only allows a value estimate for each of the triadic estimates in the data, but it also provides a validation basis with all estimates showing a trend in their behavior that logically should converge to a constant value (as the same property is evaluated each time).

In this work, we exemplify how the EWM approach effectively addresses the limitations of triadic methods, merging them into dependable point estimates. This technique streamlines and completes the computational cycle within the conventional appraisal system, enhancing the transparency, elegance and depth of the appraisal system when pinpointing value based on the simultaneous utilization of triadic methodologies. By surmounting the limits inspiring this work, we can more fully appreciate the process of pinpointing a singular value from the simultaneous use of three distinct market responses. From a conceptual perspective, acknowledging three different market values that often diverge significantly for the same property offers the analytical capability to compare, justify and explain results, rather than relying on a single method. This approach potentially equalizes the analytical and repetitive technical content in appraisal reports, much of which becomes automated with advanced technology and data, leaving less room for expert insight and knowledge. The progressive automation of appraisal steps trims costs, streamlines report format and content, and exerts pressure on the profession to cultivate more sophisticated and analytical content. This unique analytical opportunity, confined to the final step of the appraisal process, can stimulate intriguing advancements.

Reliable estimates of value are essential for a wide range of agents, including property owners, investors, cities, governments, developers, constructors, buyers, sellers and institutions that lend capital. In addition to being important for experts and these agents, proper decisions regarding real estate value are also necessary for managers at various levels and types of properties [24,25]. In addition to case-by-case evaluations, millions of properties are regularly evaluated in bulk and taxed worldwide, especially in North America where value estimates are cyclically needed every three to four years on average [26,27,28].

## 2. Literature Review

Economic agents evaluate various events or objects of desire based on their degree of expectations and information. Theories of judgment and information help to understand their subjective behaviors in forming these evaluations [29,30]. Information theory was introduced and used in econometrics by [31,32]. Statistical Mechanics sees a connection between information theory and physical entropy, where prediction is an informational matter and entropy represents the degree of uncertainty about a system’s state [33]. The discussion on the relation between information and physical entropy originally began with [34,35], inspiring [36,37] to define later information as the reduction of entropy. Entropy measures the uncertainty of a random process, providing a quantitative measure of information asymmetry [38].

Entropy calculations can be used in engineering [39], population prediction [40], linguistics [41], econometrics [42,43], biology [44,45], neuroscience [46], genetic expressions [47] or psychological subjective decisions and sensations [48,49]. Shannon’s entropy theory of information has inspired these researchers, notably in economical meaning and decision making under uncertainty [50,51]. As natural selection favors low entropy states [52], human expectations compete to attain novel information related to events or objects of interest [53]. Uncertainty in events motivates the search for information [54]. For [55], the value state is the maximum entropy state or the best choice that provides maximum information entropy. Empirical evidence clearly suggests the benefits of exploring information and entropy-based theory to better understand market behavior [56]. Once the information is disclosed, its value decreases and it becomes less scarce [57]. The entropy-based market analysis and investment theory is applied, for instance, to investment practice, asset and portfolio returns, financial time-series forecasting and estimation of manufacturing yields [58,59,60]. Elsewhere, the investor performance is shown to depend on informational advantages [61,62].

In economic behavior, the reduction of entropy is clearly a key concept. According to Schrödinger [63], a Nobel Prize-winning physicist, low entropy governs economic values [64]. Applebaum [65] similarly argued that entropy is a measure of a commodity’s scarcity and value. Georgescu-Roegen [66] was a pioneer in thoroughly exploring the relationship between economic decisions and the natural environment. His work marked the inception of what we now recognize as entropy economics. According to his research, economic value embodies properties of complexity, indeterminacy and human behavior, all derivatives of the law of entropy. He meticulously detailed the relationship between thermodynamic entropy and economic value, particularly focusing on the value of commodities, which, in the context of this work, refers to real estate value. The dynamic expressions of value through PCI observables and conventional methods were enlightened by his profound explanations and connections to thermodynamic entropy. While our focus here is on informational entropy as a tool to enhance property value measurement, it’s important to recognize that thermodynamic entropy is already ingrained in the conventional PCI observables and methods that form the basis of this work.

Though the literature provides formal definitions of thermodynamic and informational entropies (see the comprehensive work by [67]), drawing connections to real estate proves useful. Real estate properties undergo energy and material transformations due to their material constitution. Economic agents make decisions regarding property attributes (influenced by thermodynamic forces) using comparable information on PCI realizations under conditions of uncertainty (economic decisions understandable via informational entropy). The value of a property shifts in response to the quality/quantity of location attributes (e.g., a parcel of land in a humid/sunny region or a convenience store in a rat-infested commercial building) and depreciation as the building ages. PCI represent these dynamics, reflecting their economic evaluation. Economic agents not only react to thermodynamic environmental impacts, but they also strategically plan and modify the property and location attributes to align with the most probable value state (as assessed by economic agents). For example, entrepreneurs build convenient and better-structured buildings to combat thermodynamic forces, utilizing knowledge and technology to shape and reduce cost levels. This strategy creates buildings that offer better protection against weather conditions, relative to price, income, and most crucially, value state. Demand side agents will set prices (and generate incomes) accordingly, considering uncertainty as they evaluate PCI levels, and also referring to the most probable position of value state.

In the context of economic valuation, decisions regarding desired events/objects are often made in uncertain environments. To make these decisions, information or different types of observables such as PCI are used to feed personal or expert evaluation methods [68]. However, it is important that the methods optimally represent the real world and do not overfit or artificially create noise in the estimates [69,70]. Not all methods perform similarly. Data quality must be factored, and they should be compared based on cross-validation and degree of uniformity in the distribution of prediction errors as well as favor low cost and faster prediction [71,72]. A single method can perform well, but multiple methods should be used to exploit further potential of the data, which is especially important in situations of decision making [73]. In that regard, entropy is frequently used in the field of artificial intelligence for data fusion, attribute contributions and multimodal global predictions. Multimodal data fusion involves transforming information from multiple single-mode representations to a compact multimodal representation, similar to the thermodynamic process of melting a solid substance [74]. This technique is used in various fields, such as image fusion, which combines multiple source images of the same scene to create a fusion image that is more suitable for human visual perception, computer processing, or surveillance for more accurate decision making [75,76].

To integrate attribute contributions or estimates from multiple methods into one indication for decision making, it is necessary to rank and weight them appropriately. Various methods have been developed for this purpose, including the eigenvalue method, which captures rank orders [77], conjoint analysis [78] and analytical hierarchy process (AHP) [79]. Synthetic indicators of the residual distribution by Bayesian Information Criterion (BIC) and the Akaike information Criterion (AIC) are widely used as model selection criteria [80,81]. Other methods that have been developed for this purpose include the quadratic extrapolation method [82], the Panzeri-Treves Bayesian estimation [83], the Best Universal Bound estimation [84], the Nemenman-Shafee-Bialek method [85] and entropy-integrating fuzzy ranking [86]. More recent methods using statistical copulas have been developed [87]. Each of these methods has its advantages and disadvantages [88].

Entropy calculations are known to be versatile and compatible methods, particularly in assigning priority and importance ratings of attributes/methods [89,90]. Entropy methods are commonly used to reduce uncertainty in MCDM [91]. In MCDM, researchers utilize Multi-objective optimization (MOO) weights of importance to be assigned to functions for simultaneous optimization problems [92]. Researchers have proposed different ways to solve optimization problems to support a decision maker in finding an optimal or equilibrium state [93,94]. The MOO techniques utilize response weights in their process of converting multiple responses into a single response. In this process, decision-makers must understand the true meaning of weights and their computations. The involvement of the weights of importance in MOO is crucial in the entire optimization process and ultimately operates at the last step, where they are used to conclude results for a final decision. Assigning weights to responses in MOO can be done in different ways, such as (a) equal assignments [95], (b) subjective weights from judgment or personal opinion of the decision-maker (an expert) including Ranking weighting [96], Allocation of points [97], Trade-off [98], Pairwise comparison [99], LSQ method [100], Eigenvector method [101] or Delphi method [102] and (c) objective weights from mathematical models or algorithms using observables without the involvement of the decision-maker. These include the Entropy method [103], Vertical and Horizontal method [104], TOPSIS and Principal component analysis [105], Variant coefficient [106], MOO method [107], and so on. Any combination of these can be used.

Several studies have explored and integrated the potential of the entropy method in various ways and goals in real estate analyses [108]. Zhou et al. [109] extended entropy principles to real estate risk assessment and weighting based on Shannon’s entropy. Lam et al. [110] utilized the entropy method to find weights of the selected variables affecting property price and compared results with multiple regression analysis (MRA) and Artificial Neural Network models’ outcomes to notice that entropy method performs better. Chan et al. [86] proposed the fuzzy and entropy method to obtain the final ratings of the customer needs. Gnat [91] utilized the entropy approach to define the degree of homogeneity of properties in given sectors. Lam et al. [111] demonstrated that the integration of entropy and ANN can account for variance in the housing price determinants and improve forecasting progress. Salois and Moss [112] examined the change in information in net value added to farmland and values over time, as well as the relationship between the two. Results indicate that new information increases the entropy in the short term but reduces the entropic signal in the intermediate term. This loss in short-run information may be the result of random shocks, which do not persist or produce responses to market changes. Sekaran [113] suggested that different cultures reacted differently to scaling issues, and used the five-point scale to capture the meaning of the subjective judgements.

## 3. Value State Balance

Value holds profound significance and application in nearly all fields, with particular emphasis in economics [114,115]. However, the interchangeable use of the price, cost and income (PCI) components often leads to ambiguity in its definition and a lack of a coherent framework to extract their true meaning in relation to value [116]. The challenge of measuring value is a fundamental concern and, unlike entropy, it involves difficulties in its methodology, perhaps due to terminological confusion [117,118]. Various perspectives have been proposed by different schools of thought, primarily in economics [119,120,121].

To elucidate the basis of value, authors traditionally differentiate between use value and exchange value [122,123]. Classical thinking proposes that value derives from the cost of production agents (supply), whereas the neoclassical viewpoint considers the utility of demand-side agents. Marshall [5] merged these objective supply-cost definitions of value with the subjective demand-price propositions to account for the quantity of commodities. Subsequently, a debate ensued about replacing the subjective use value with the objective exchange value [124,125]. Post the industrial revolution, mainstream economics championed the concept of exchange value, given its practical application and the ability to use PCI observables as sufficient substitutes for value. Researchers in other fields typically define value in reference to this dominant view of exchange value, perceiving value as a function of scarcity and (marginal) utility. Value is also defined as a blend of both use and exchange values [126].

The perspective of exchange value interprets the value of commodities by their observed PCI in the market [127,128]. In contrast, use value pertains to individual emotions and subjective expectations when processing information [129]. These expectations transition into exchange values once they are expressed in the market via different forms of PCI transactions. PCIs are continuously updated and serve as informational references to shape new expectations. Unlike subjective personal-use values, PCI are determined through negotiation, always referencing the most probable position of value state.

PCI involves a comparative process of negotiation and multi-criteria decision making, taking into account property attributes in relation to their impact on the market. Supply and demand agents negotiate and decide on selling price levels, cost of production entrepreneurs and income stream investors, all referring to the position of value as different bases of information. For instance, the price is an observed (or past) expression of value state resulting from an evaluative and comparative decision-making process by subjective buyers and sellers. The price does not equate to a value state; it serves as its imperfect reference (used in its approximation like in the evaluation system). The same decisional process between two different types of economic agents, observed for different manners and in two additional different contexts, leads to two other observables of cost (related especially to an ongoing production process of the same commodity in the present) and income (related to the same commodity in postponement/projections and strategic planning of future streams of periodic incomes).

**Figure 1:**
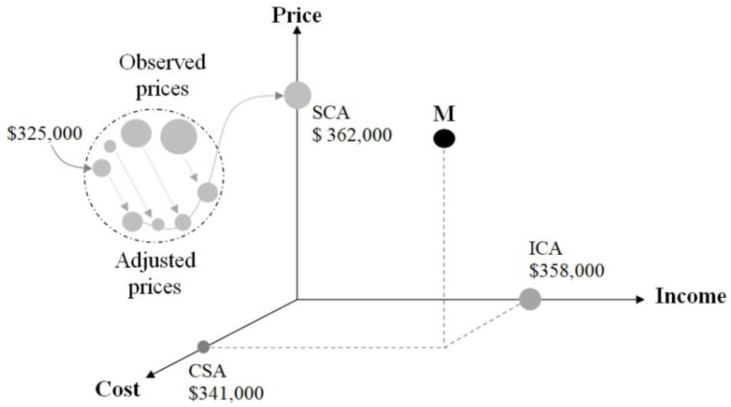
PCI negotiation steps between buyers and sellers.

Consider the negotiation contexts for a residential property, as depicted in Figure 1. PCI observables, which represent past, present and future comparison processes, enable agents to assign a value to the same property on the market. Essentially, these PCI responses, acting as empirical expressions and observables of the value state, facilitate the assessment of that value state. In the early stages of negotiation, the agents don’t have an exact PCI figure for the property; they rely on their personal judgment, observable PCI’s and attributes of similar properties in the same market. For example, a buyer might be willing to pay a high price while the seller might propose a significantly lower price. These proposed prices will change throughout the negotiation process, eventually culminating in an agreed price of $325,000, as demonstrated in the example.

The final negotiated price provides one dimension of the property’s value state, but there are also the cost and income dimensions that, when taken together, give a more comprehensive perspective on the most probable position of the market value of the property [130]. Importantly, the price reflects the past appreciation of the property’s utility attributes within a buying and selling context, with reference to the value state. This value state is not isolated from the contexts of the cost of production and the income generated from the exploitation of the property. The cost dimension takes into account the present or ongoing charges of interest, salary and rent, which reward the production agents of capital, labor and natural resources (such as land). Lastly, the projected future income streams of the property are accounted for when approximating the value state. As complementary sources of information in multi-criteria decision-making contexts, considering these factors simultaneously helps to better pinpoint the most probable market value (represented by the “M” point in Figure 1) of the property in question.

The process of adjusting, either upward or downward, in the appraisal system is crucial to ensuring that the attributes and PCI estimates of comparable properties align closely with those of the subject property. What’s important to note about this adjustment process is that all necessary modifications have been implemented to make the property comparable; hence, any potential variations have been accounted for. At this final stage of the evaluation process, the expert also has to make a final decision on the most probable market value of the same property, which serves as the best approximation of the value state. Each valuation method carries its own advantages, disadvantages and unique characteristics, which depend on various factors. These include the type of property (certain methods are more suitable for specific property types), the availability of data, the extent of required computations/adjustments and the current state of the market.

The appraisal process can use three concurrent methods, each supported by PCI observables, respectively: the *Sales comparison approach* (SCA), the *Cost summation approach* (CSA) and the *Income capitalization approach* (ICA). The SCA is a more direct way of estimating the market price of a property, as it relies on the observed prices of comparable properties in the market. When the data from the market is poor regarding price and income, the cost method remains an alternative for the market value estimation of generally unusual properties. On the other hand, the CSA cannot estimate land value and may be influenced by cost depreciation estimation. The ICA is preferred for properties that generate stable income and have reliable market indicators like a stabilized cost and income, but it relies on projected and hypothetical data that are uncertain and derived from other estimations [131,132].

It is generally assumed that the application of the three methods simultaneously would result in three identical or closely similar values for the same property. In the example we use below as a practical demonstration, the SCA, CSA and ICA provided estimates of $362,000; $341,000 and $358,000, respectively. The expert must then decide on the final market value of the property based on local data at a specific date. The expert may suggest the SCA estimate of $362,000 because the data is recent and abundant on this market, in comparison to the results of the two other methods. An average of $354,000 is another alternative of value response of the subject, and a weighted average based on the reliability of each method would indicate a value of $357,000, assuming weights of 0.6, 0.2 and 0.2 by the expert’s intuition and knowledge of the market. These are the two known alternatives to conventional PCI evaluations [8,133]. Practitioners may prefer either an inclusive simple and weighted average or an exclusive approach depending on the reliability and type of information available [134]. However, there are no established guidelines for selecting the best approach in the literature and practice.

In the considered example, the variances between the estimation methods may vary depending on the subject of inquiry. In these instances, all the potential of data, parameters, computations and approaches have been involved in order to bring all the necessary adjustments. The same subject is evaluated by the same method with low internal variance, yet the values between methods often do not converge. It is challenging to logically explain and improve prediction processes and approaches in such situations, even when using competitive triadic approaches and following a thorough evaluation process. This poses a major concern for experts and decision makers because the most critical step in the evaluation process, the decision-making moment, becomes void. Consequently, PCI expressions by economic agents and the considerations that lead to market values by experts cannot be confidently used in MCDM. Even if estimates converge, the value state largely retains inefficiencies using the current framework of classic evaluation.

## 4. Entropy Weights Method

The Entropy weights method (EWM) works well across diverse MOO problems in MCDM [2]. Shannon and Weaver proposed EWM in 1948 [36] that have been emphasized by many other authors in further developments [135]. Since then, various advancements have been made in the methodology of EWM in different fields, such as fuzzy entropy method, cross-entropy method, Grey entropy technique, intuitionistic fuzzy entropy weights, etc. However, research is still ongoing to expand the method. The EWM offers several advantages, such as the ability to compute relative weights of responses in a simple, unbiased way, successfully assess indicators, appropriately identify divergence of responses and calculate their weights, suggest the requirement of supplementary information, compute effectiveness and advantage/cost responses, account for the weak impact of unusual attributes, and deliver more precise outcome with more different coefficient values for responses. Additionally, the EWM is suitable for the entropy strategy to handle the fundamental disagreement between the responses in decision making [136]. Despite the various benefits, some possible downsides of the EWM can be related to appropriate problem sizing [137], a lack of specialist verdict in computed weights and a sole focus on entropy values. Additionally, EWM does not provide any participation in the designer’s preferences, and its discretion in decision-making has been reported as it pays no attention to rank discrimination [138].

To compute uncertain information (Entropy), probability theory is utilized. The EWM works on the principle that superior weight indicator information is more constructive than the lower indicator information [139]. This method involves deciding objectives (decision matrix), calculating the normalized decision matrix, the probability of the attribute/response to take place, the entropy value of attribute/response, degrees of divergence (average information contained) by each response and then the entropy weight.

In this research, we propose that value has a basis that can be explored and approached in the entropy framework. We present the basis of this approach in the following sections with equations of the EWM. Numerous alternative techniques can be specified to integrate and improve the real estate evaluation process within the general framework of the EWM. It’s important to note that our approach picks up where traditional PCI evaluation methods leave off, offering a singular market value. This market value is an approximation derived from averaging three different market values, which are based on SCA, CSA and ICA. While this averaging process provides a valuable perspective on a property’s market value, there still exist variances between triadic approaches. To mitigate these variances, we strive to introduce an objective weighting using the EWM. Our weighting process begins by utilizing three PCI market values, under the assumption that all the necessary adjustments have already been made in their detailed computations. The market value produced by the EWM is expected to be more objective and efficient, provided its results converge to a singular value state over a set number of trials (in our case, ten trials per three different methods). We will delve deeper into this in the following section, but first, we will present our approach as follows:

The decision matrix of data is detailed in Equation (1) in which every row of decision is allotted to one experiment and all columns to one determining variable (here triadic PCI estimation methods are considered as evaluation responses or attributes). Accordingly, the elements e of the PCI evaluations $e_{\mathrm{ij}};i=\left\{1,\;2,\;3,\ldots ,n\right\}$ where n is the number of experiments and $m=\left\{1,\;2,\;3,\ldots ,m\right\}$ represents the response number in the matrix.

(1) $$\mathrm{DM}=\left[\begin{array}{ccc} P_{11} & C_{11} & I_{11}\\ P_{21} & C_{21} & I_{21}\\ P_{31} & C_{31} & I_{31}\\ \ldots & \ldots & \ldots \\ P_{\mathrm{nm}} & C_{\mathrm{nm}} & I_{\mathrm{nm}} \end{array}\right]$$

The linear normalization technique is utilized to make the experimental data of DM dimensionless due to several units of the variables. Equation (2) is used for beneficial attributes, i.e., those having positive impacts between them and value.

(2) $${\mathrm{NDM}}_{\mathrm{ij}}=\frac{e_{\mathrm{ij}}}{{\mathrm{Maxe}}_{\mathrm{ij}}}$$

The above equation assumes that evaluation response variables are in positive relation between them and value. In case a variable among them negatively affects the value state, then the Equation (2) becomes ${\mathrm{NDM}}_{\mathrm{ij}}=\frac{{\mathrm{Mine}}_{\mathrm{ij}}}{e_{\mathrm{ij}}}$.

The probability of each variable $\left({\mathrm{Pr}}_{\mathrm{ij}}\right)$ is computed by the Equation (3), with a range ${\mathrm{Pr}}_{\mathrm{ij}}\in \left[0,1\right]$.

(3) $${\mathrm{Pr}}_{\mathrm{ij}}=\frac{{\mathrm{NDM}}_{\mathrm{ij}}}{\sum_{i=1}^{n}{\mathrm{NDM}}_{\mathrm{ij}}}$$

The following Equation (4) is utilized to calculate the Entropy $H_{\mathrm{ij}}$ of the X probabilities ${\mathrm{Pr}}_{\mathrm{ij}}$.

(4) $$H_{j}=H\left(X\right)=-E\left[\log_{b}P\left(X=x_{\mathrm{ij}}\right)\right]=\sum_{i=1}^{n}{\mathrm{Pr}}_{\mathrm{ij}}\log_{b}\frac{1}{{\mathrm{Pr}}_{\mathrm{ij}}}=-\sum_{i=1}^{n}{\mathrm{Pr}}_{\mathrm{ij}}{\;\log}_{b}\;\left({\mathrm{Pr}}_{\mathrm{ij}}\right)$$

Note that $\log_{b}\left(n\right)$ in the entropy equation above represents the Maximum of the entropies of responses PCI estimates depending only on the uniform (equiprobable) distribution of n observables.

The ratio between $H_{j}$ and $\log_{b}\left(n\right)$ in Equation (5) represents the contribution fraction of attribute j. In effect,

(5) $$Y(-\sum_{i=1}^{n}{\mathrm{Pr}}_{\mathrm{ij}}{\;\log}_{b}\;\left({\mathrm{Pr}}_{\mathrm{ij}}\right))$$

In this equation, $Y=\frac{1}{\log_{b}\left(n\right)}$, which is a stable expression. The Equation (5) is also used to calculate the divergence of the entropies from the maximum by the Equation (6):

(6) $$\mathrm{abs}\left[1-\left[Y(-\sum_{i=1}^{n}{\mathrm{Pr}}_{\mathrm{ij}}{\;\log}_{b}\;\left({\mathrm{Pr}}_{\mathrm{ij}}\right))\right]\right]$$

It must be remembered that the effect of the logarithm in the entropy formula is to transform multiplication into addition and division as indicated in the Equation (7).

(7) $$\mathrm{Pr}\left(X,Y\right)=\mathrm{Pr}\left(X\right)P\left(Y\right)\;\to \;H\left(X,Y\right)=H\left(X\right)+H\left(Y\right)$$

When non-additive entropies are involved, as we assume to be the case in this work, there is a certain dependence between explicative/determining variables. It is imperative to identify these non-additive mutual entropies and measure them [140]. We did this using the Havrda-Charvat-Tsallis entropy, also known as q-entropy or Tsallis index [141,142]. A generalization of Boltzmann entropy, it is well established for systems that are as precise and fundamental as the foundations of Boltzmann entropy [143]. We estimate this entropy measure by the following general Equation (8) to remove redundancies in the calculated entropies.

(8) $$S_{q}\left(A,B\right)=\left(1-q\right)S_{q}\left(A\right)S_{q}\left(B\right)$$

The mutual information defined by the Equation (8) represents the degree of divergence from the linearity of the entropies. This equation applies in the case of attributes with positive impacts on value. When the impact is negative between attributes, it becomes $S_{q}\left(A,B\right)=\left(1+q\right)S_{q}\left(A\right)S_{q}\left(B\right)$. The entropic index q in D-dimensional space is computed using the following Equation (9):

(9) $$q=\frac{D\left(n-1\right)-4}{D\left(n-1\right)-2}$$

where D represents dimensional space (number of determining variables) and n the number of experiments. Equation (10) is utilized to compute the degrees of divergence (${\mathrm{Div}}_{j})$, and Equation (11) obtains the entropy weight $E_{\omega }$ of the jth response.

(10) $${\mathrm{Div}}_{j}=\left|1-{\mathrm{En}}_{j}\right|$$

(11) $$E_{\omega j}=\frac{{\mathrm{Div}}_{j}}{\sum_{j=1}^{m}{\mathrm{Div}}_{j}}$$

The larger the ${\mathrm{En}}_{j}$ is, the greater the differentiation degree of index I is, and more information can be derived. Hence, higher weight should be given to the index.

From the divergence estimated by EWM, we can deduce the weights given to the PCI approaches. The overall value V of a subject property in Equation (12) is estimated based on these weights ω, multiplied by the maximum of PCI evaluations, respectively. The use of maximums is because the entropy comparison was measured against $\mathrm{Ln}\left(n\right)$, which is the maximum.

(12) $$V=\left(\omega_{P}\times {\mathrm{Max}}_{P}\right)+\left(\omega_{C}\times {\mathrm{Max}}_{C}\right)+\left(\omega_{I}\times {\mathrm{Max}}_{I}\right)$$

The steps following the equations above lead to a final value by the fusion of conventional triadic PCI estimates operated by the EWM. While satisfied with the specification of equations, we would like to see the case-by-case evaluations, also using the Equation (12), and verify if their estimates are stable.

In the method described above, we compared the global entropy of PCI estimates with respect to the maximum $\ln\left(n\right)$. Everything in this method comes down to multiplying the global entropy of PCI by $1/\ln\left(n\right)$ that [135] defined by Y. As this Y term is uniformly applied to all cases, we adapt it for individual cases as $Y_{i}=D\left(p_{i+1}||I\right)$, which is the following Kullback and Leibler [144] divergence measure (also denoted as KL divergence).

(13) $$D\left(p_{i+1}\|p_{i}\right)=-\sum_{i=1}^{n}p_{i}\ln\frac{p_{i+1}}{p_{i}}=-\sum_{i=1}^{n}p_{i}\left[\ln\left(p_{i+1}\right)-\ln\left(p_{i}\right)\right]=\sum_{i=1}^{n}p_{i}\ln\left(p_{i+1}\right)-\sum_{i=1}^{n}p_{i}\ln\left(p_{i}\right)$$

In the detailed calculation, KL divergence considers the difference between one experiment in comparison to the subsequent one. Integrating this specification in the EWM framework allows individual weights, adjustments, and an entropic value per triadic PCI estimate from the conventional appraisal system. This case-by-case estimate most importantly turns out to be a comparable basis for validation. The main condition of validation, in this context, is the trend that the value states converge for the same property. This makes sense, as the utility determining value state attributes are the same (after all necessary adjustments have been satisfied).

## 5. An Empirical Demonstration

We conducted an empirical demonstration to show the applicability of the conceptual foundations of the entropy method in understanding the meaning of the value state and its connections to the EWM. To illustrate this, we considered the real estate appraisal system and data on a single-family property taken from estimations in [13]. The property was evaluated by Özdilek using conventional triadic SCA, CSA and ICA. This data was originally gathered and made available online by Robert Shiller, a Nobel Prize-winning economist, and covers a period of 129 years from 1890 to 2018, also used in his works such as “Irrational Exuberance” [145]. As shown in Table 1, we considered the subject property that is estimated in detail in 2018 (estimation response no. 8), as well as its 9 other estimates (each representing almost 13 years of the market) for the same period considered by Özdilek.

**Figure 2:**
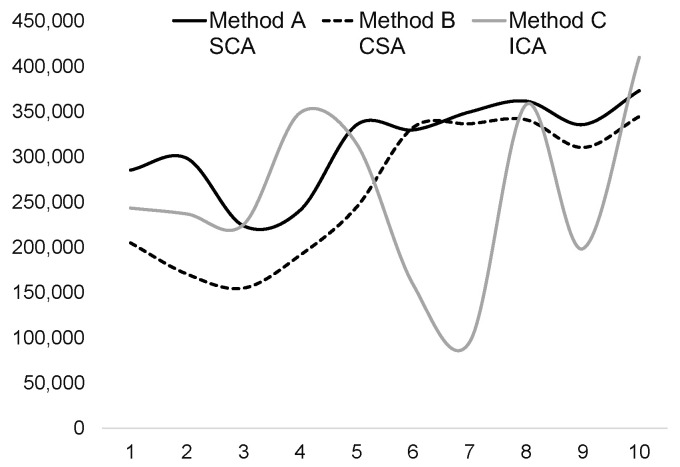
Subject property estimates by triadic appraisal methods.

We used a decision matrix consisting of these ten experiments by three types of PCI responses to evaluate the value state of the subject property. The final step of this evaluation process was to arrive at a decision about the market value of the property to enable more decisions from multiple economic agents. As shown in Table 1 and Figure 2, we encountered significant divergence in the results for the same property, despite making all the necessary adjustments within the traditional PCI methods. Ideally, this table should contain thirty identical or very close market value estimates across three different approaches. However, the market value varies significantly between $95,488 and $410,248. If it is the same property, and all the required adjustments have been made, what could be causing these discrepancies? According to these results, we would tend to propose a response where PCI evaluations converge to a similar value as is the case with the 8th, 10th or even 1st response evaluations. It should be noted that there are still divergences between these cases. Despite considering all possible attributes of the property, market conditions and PCI evolutions over time, there were still significant variations among the conventional methods, requiring further clarification on the source of variance.

We propose that these variations arise from the differing degrees of reliability embedded within traditional PCI methods, necessitating a final, objective adjustment in the last evaluation step, which we accomplish through the EWM. We also consider that these variations are influenced by the fact that economic agents negotiate and make decisions based on PCI observables, influenced by their personal knowledge, needs, goals, and various other factors (beyond property attributes) that emerge in different PCI transaction contexts. Additionally, the competition between PCI, during which the referential position of value state of the subject is considered by economic agents, might also contribute to the observed variations.

In practice, only one of these responses is estimated, and even so, the estimation is mostly limited to two of the triadic conventional approaches (within the same response). This difficulty results from the quality of input data, not the evaluation methods used. For instance, for the 8th response, the expert can approximately assume a value of $362,000 by the SCA for the property by primarily considering price estimates, and while excluding estimates using CSA and ICA. The expert can also factor in the estimation of the cost method, but this involves the difficulty of weighing two estimations. In this case, the common estimation (response) would be either a simple average with equal weights for both methods ($351,000) or different weights—for instance, 60% for the SCA and 40% for the CSA (in which case, the final value estimate would be $353,000). As long as different weights of importance can be attributed to the three methods, then the expert can suggest, for instance, 60% for the SCA, 20% for the CSA and another 20% for the ICA (leading to a final market value estimation of $357,000). The problem here is the subjective weights of importance, which are attributed in this final step of evaluation and ensue value estimates, involving measurement bias and error. The real issue at this final step is thus finding the appropriate weights of these responses in triadic methods.

As part of the intermediary steps of the EWM, we operated a linear normalization technique to make the experimental data of Table 1 dimensionless. Equation (2) is used for a beneficial response, i.e., for positive impact on value state of the responses, whereas if these factors act negatively on value, non-beneficial responses can be used. Accordingly, SCA and CSA are normalized based on Equation (2) of positive impacts; the ICA is rather based on its non-beneficial version as ICA is negatively correlated with the two other ones as reported in Table 2. This table contains another normalization process presented in Equation (3) to generate probability of the responses, necessary for the computation of the entropies contained in the last three columns of Table 2, based on Equation (3).

Table 3 summarizes the entropies obtained for PCI approaches. Using the 10 responses per method in our data, the Y term results in a value of 0.4343. Multiplying this term by the sum of the entropies of each approach yields the results of Equation (5), with maximum values approaching 1 when the entropies converge per approach. This indicates that the previous steps of attribute and price adjustments in the conventional evaluation system have efficiently led to the same market value. However, as shown in Table 2 (three last columns), variations in entropy measures can detect inefficiencies or uncertainties, allowing for a final entropic adjustment. We also need to include the interaction term from the mutual entropy.

The most important step in this study is the computation of divergence, which has two critical applications. Firstly, it determines the reliability of each method; secondly, it allows us to estimate the adjustment amount needed to reach a final value for the subject. The less an approach requires adjustment at this final stage, the higher its priority in the final value estimation. Following the steps of equations specified in Table 3, the ICA requires the highest degree of adjustment, which is $165,800. Adding these adjustments yields a final market value of $380,000 (rounded) in the last step of computation.

The estimation of a stable and final value state from the fusion of the triadic conventional methods based on their respective weights derived using the entropies in Table 2 is already satisfactory. This is consistent with what we have already observed in two stable (constant) points of value states at the initial point estimates for cases no. 8 and 10 in Table 1. The logic behind the confidence in these values is that if the same or similar properties are evaluated, we should expect that different methods result in converging values, i.e., towards the region of a pointwise reference, which is near the value state. EWM efficiently and quickly allows us to globally find that value state in this work.

We could be tempted to add each of the adjustments to the diverging estimates at the last step of the conventional estimates in Table 1, yet it is unnecessary since the entropy evaluation has already provided a global constant value of $380,000 for all the potential responses. For the sake of completeness and validation (a single estimate of market value based on the EWM does not guarantee that value state is effectively pinpointed), individual estimations of value state can still be provided instead of a global fusion between all the estimate responses as an extension and improvement of the EWM. For instance, adding $111,937 of the entropic adjustment to the first four cases of the SCA will bring them closer to other estimates, meaning that they all converge on the same market value estimation since the subject is identical. The same can be done for the two other methods, which will bring entropic weight measures to the same level (three last columns of Table 2), with cascading effects on the following results in Table 3 where we observe that we drive entropy results to identity.

Starting from the same entropy estimates of Table 2 (three last columns), the following Table 4 shows new estimates of entropy weights for each approach on a case-by-case basis, which we distinguish in a second EWM 2 (for individual estimates, denoted by each row). Accordingly, the weights in the three last columns are not only different for each approach, but also per response. Their sum allows the estimation of the value of the subject in Table 5.

The enhancement of EWM 1 by introducing a novel approach in EWM 2 enables us to produce case-by-case estimates while maintaining a comparable basis of results. This was achieved by incorporating the number of experiences in the entropy approach in a unique way. Rather than using a fixed number of experiences, we varied them from 1 to 10, for each case assuming that the information gain is highest with the first experience and subsequently decreases when new experiences are added, reducing the level of accessible information. The KL divergence has proven to be effective in meeting this requirement, as evidenced by the fact that the results for the 10 experiences are becoming closer to each other on a case-by-case basis and between the fused 10 final estimates.

**Figure 3:**
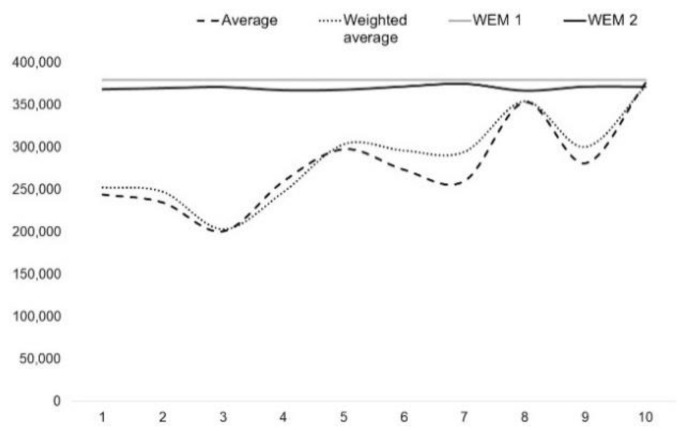
Results of comparative methods.

The Figure 3 on the right side shows the results of the WEM 2, which are highly promising, particularly considering that they are close to the global estimate from the WEM 1 of $380,000. Furthermore, the values from WEM 2 outperform the average values of the conventional approaches even if some expert weights are incorporated, which are slightly better than simple averages. The results of WEM 1 and WEM 2 demonstrate the potential of entropy to explain and predict value states. The results of the WEM 2 further establish entropy as a highly compatible tool that can be seamlessly integrated into the appraisal system, significantly enhancing its methods, particularly at the evaluation’s conclusion. By integrating the EWM, we can propose a more reliable and objective market value estimate using PCI methods, providing the most credible methodology to date. This confidence stems from the fact that we offer multiple proofs of evaluations, which show a clear trend and validation that aligns with a linear projection in Figure 3. This projection is significant evidence given that the subject under evaluation remains consistent, and thus, constant values are expected. Entropy promises to propel the real estate appraisal system’s power and quality to new heights.

## 6. Conclusions

Value-state information disclosure acts on the expectations and decisions of economic supply and demand side agents. Because of its probabilistic and dynamic nature, value state complicates the process of personal and expert evaluation. Existing PCI concepts, observables, and methods within the framework of the worldwide appraisal practice go through many technically detailed steps of computations and adjustments within each PCI approach. Though this appraisal system can produce valuable triadic estimates (competing opinions) on the value state of a specific property (the “subject”), the practice predominantly depends on a single approach. Usually, two out of the three PCI are considered, but it’s uncommon for all three to be utilized. Unfortunately, this introduces variance in value state estimations. PCI concepts, observables and approaches considered simultaneously provide a better estimation of the value state. This is the most crucial step of the appraisal system where the analytic and scientific acts of measuring value state begin, assuming that the technical processes in the previous steps of evaluation within each approach have been considered closely.

In our current evaluation system, we can opt for either an exclusive or inclusive practice in providing a final point value state estimate for a subject property. In the exclusive practice, the prevalent argument is that one of the PCI approaches more accurately represents the value state due to richer active information. Alternatively, the inclusive practice could involve two or three approaches simultaneously, either assuming equal or variable weights in approximating the value state. In both cases, it’s essential for experts to provide objective, justifiable computations and explanations for the simultaneous weighting and adjustments of PCI approaches. Our work initiates from this final step of the evaluation system, where measuring the value state is the most challenging aspect. To overcome these limitations, we introduce and utilize the Entropy weighting method (EWM) in the final step of the evaluation system as a primary value measure in conjunction with conventional PCI methods.

The use of the EWM offers an important advantage over traditional methods, such as average or weighted average provided by subjective experiences. The EWM objectively assesses the weight of each method and simultaneously fuses the market value estimates of conventional approaches for a reliable pointwise approximation of the value state. To systematize its integration, we empowered EWM capacity by considering the treatment of mutual entropy and operationalizing the process of adjustments of conventional estimates in the ultimate step of evaluation. The results we generated in this work with the help of entropy measure are satisfactory and demonstrate its accurate application in property estimation. Based on the entropy principles and connections to value state, EWM calculations finally provide a simple, versatile and integrative framework that clearly improves traditional methods, in accurately providing pointwise predictions as demonstrated in this paper. This innovative alternative will benefit numerous stakeholders, such as property owners, private investors, institutions and governments by reducing bias and errors in value state estimation. Enhancing the core of the evaluation practice by introducing an additional step boosts the robustness of the evaluation system for experts and increases public confidence in the system.

Working with the results of the EWM, we remarked that it has the potential to further improve the current system of evaluation. In addition to this global approach that estimates a fused value for all the responses in the whole data, we also tried the EWM in case-by-case evaluations, adjusting each imperfect estimation of conventional approaches. For that, we needed to adjust the EWM and integrate the Kullback-Leibler divergence in relative entropies of each case in the data. This is an important technical contribution of our work as it not only allows a value estimate for each of the triadic estimates in the data, but it also provides a validation basis with all estimates showing a trend in their behavior that logically should converge to a constant value (as the same property is evaluated each time). This enhancement enables experts and cities, especially during mass evaluations, to justify the objectivity and reliability of the results.

Real estate market prices are often established through negotiations between parties and possible intermediaries. Integrating EWM into conventional PCI methods to derive a point estimate might limit the flexibility of real estate markets, potentially reducing transaction volumes and long-term value states. Assuming the improved reliability of estimates by the PCI/EWM combination leaves less room for “entropic behaviors” from supply and demand side agents, creating higher risks but also opportunities. PCIs are compared and observed in the market, and future negotiations will likely become more adept over time, converging towards more stable market values. This hypothesis that emerges from the results of this work certainly deserves to be explored in future research.

These global and case-by-case estimation results show that, unlike conventional methods, entropy can objectively detect and measure value state beyond what PCI attribute adjustments can do in the conventional process of estimations. In that respect, entropy more accurately considers the interplay between expectation and information in value states that are out of the reach of conventional methods. We have observed that there are certain subjective and unexplained aspects that exist beyond the variability of attributes that are not entirely captured by the variability of PCIs. Entropy accounts for PCI variance implicit in expert estimation, providing a wider and more appropriately approximating market value. From this perspective, this work illustrates the shared properties between entropy state and value state, which are vital considerations in Multi-criteria decision making (MCDM). We believe that illuminating these connections encourages the development of valuable research in this field.

There is something more than what is explorable through these attribute adjustments and PCI information such as the explanation captured by mutual entropy on the dynamics of competing PCI formation, which are alien to conventional processes of individual attribute and PCI method adjustments. In fact, PCI conventional methods can consider all types of internal adjustments, quite well exhausting the potential information in each PCI estimate. Starting from these adjusted final estimates (in which there were significant differences in several cases despite the consideration of the same property), entropy measures of value state show that there is significant incentive for more adjustments with EWM integrating mutual information correction and KL relative divergences. This mutual information contains the effects of interplays between competing PCI sales, cost production, and income exploitation agents under the governance of value state. What that means is that the value state should converge to a stable value state for every estimation of all potential methods for the same property (everything becomes the same or similar, considering decisions of economic agents are sufficiently rational and properly adjusted). If that theoretically assumed convergence varies significantly, as was the case in our data of conventional estimates, it is because of their neglect of PCIs attempting to mutually reconcile their reward freed from the value state to which they all refer.

Decision theory considers information as decreasing uncertainty, and entropy can be used to reduce uncertainty in the evaluation process in economics, especially within the field of real estate appraisal. Entropy naturally reflects the thoughts of economic agents who not only see the observable physical and economic indicators, but also the information they deduce as perceived value. It’s notable that entropy is closely related to the probabilistic state of value, with its mechanisms of expectations and information. It attracts the undisclosed (uncertain) portion of information in the object or event of desire, and these desires are human and non-algorithmic in nature. The desire to disclose this value is the source of motivation. As added information is revealed, the attraction to value diminishes until it disappears entirely. Expectation and information follow opposite patterns, with expectation decreasing as information is revealed, while information increases up to a maximum level where it equals the constant value for a given type of event or object. This interaction mirrors the properties of entropy, which also combines expectations and information. Information and entropy transform raw data, like PCI, into the essence of value, potentially addressing the limitations of statistical methods.

In conclusion, this article wrestles with the question: Is the value of real estate an objective or subjective concept? Arguments for both perspectives are presented. On one side, there are compelling arguments suggesting objectivity in the value concept, determined by wider market agreement converging on a narrow range of values. However, from another viewpoint, information entropy indicates value as a subjective notion, one that cannot be completely defined. Analogously, our approach resonates with the method of value determination utilized in quantum economics [146]. An appropriate comparison can be drawn to Heisenberg’s uncertainty principle. The principle implies that it is impossible to precisely pinpoint a value state; instead, identifying a narrower region where an economic value state lies would be adequate considering the precision of the quantum measurement. The value is indeterminate at the onset; hence the price of the property cannot be predetermined. The value and PCI can only be measured accurately through a transaction instead of being determined beforehand in a more objective manner. Given that transactions involve the exchange of money, money must serve as the measurement tool in the markets. Therefore, we can say that the precise value of real estate transitions through subjective singular independent valuations, and transition to objective universally agreed upon valuations via transactions.

## Figures and Tables

**Table 1:** Subject property estimates by triadic appraisal methods.

Response	Method A SCA	Method B CSA	Method C ICA
1	285,425	204,963	243,519
2	298,389	170,446	236,968
3	223,802	154,940	225,295
4	240,973	191,293	348,844
5	335,451	244,491	314,565
6	329,842	332,542	158,925
7	349,707	336,634	95,488
8	361,660	341,000	358,000
9	335,700	310,398	198,564
10	373,234	344,628	410,248
Average	313,418	263,133	259,042
Maximum	373,234	344,628	410,248
Minimum	223,802	154,940	95,488

**Table 2:** Normalized probability decision matrix and entropies.

Exp. No.	SCA Normalized	CSA Normalized	ICA Normalized	SCA Norm. Probability	CSA Norm. Probability	ICA Norm. Probability	SCA Entropy	CSA Entropy	ICA Entropy
1	0.765	0.595	0.392	0.091	0.078	0.090	−0.218	−0.199	−0.216
2	0.799	0.495	0.403	0.095	0.065	0.092	−0.224	−0.177	−0.220
3	0.600	0.450	0.424	0.071	0.059	0.097	−0.188	−0.167	−0.226
4	0.646	0.555	0.274	0.077	0.073	0.063	−0.197	−0.191	−0.173
5	0.899	0.709	0.304	0.107	0.093	0.069	−0.239	−0.221	−0.185
6	0.884	0.965	0.601	0.105	0.126	0.137	−0.237	−0.261	−0.273
7	0.937	0.977	1.000	0.112	0.128	0.228	−0.245	−0.263	−0.337
8	0.969	0.989	0.267	0.115	0.130	0.061	−0.249	−0.265	−0.170
9	0.899	0.901	0.481	0.107	0.118	0.110	−0.239	−0.252	−0.243
10	1.000	1.000	0.233	0.119	0.131	0.053	−0.253	−0.266	−0.156
SUM	8.397	7.635	4.377	1.000	1.000	1.000	−2.290	−2.262	−2.199

**Table 3:** Calculations and value estimation.

Fomulas and Calculations				Sum
$\mathrm{Hj}=\sum_{i=1}^{n}{\mathrm{Pr}}_{\mathrm{ij}}\log_{e}({\mathrm{Pr}}_{\mathrm{ij}})$	−2.2905	−2.2619	−2.1992	
$Y=\frac{1}{\log_{e}\left(n\right)}$	0.4343	0.4343	0.4343	
$\mathrm{Contribution}\;\mathrm{in}\;\mathrm{fraction}=-Y\sum_{i=1}^{n}{\mathrm{Pr}}_{\mathrm{ij}}\log_{e}({\mathrm{Pr}}_{\mathrm{ij}})$	0.9947	0.9823	0.9551	
$S_{q}\left(A,B\right)=\left(1-q\right)S_{q}\left(A\right)S_{q}\left(B\right)\;$	0.1662	0.1648	−0.1614	
${\mathrm{Div}}_{j}=\mathrm{abs}\left[1-\left[Y\left(-\sum_{i=1}^{n}{\mathrm{Pr}}_{\mathrm{ij}}\log_{e}({\mathrm{Pr}}_{\mathrm{ij}}\right))\right]\right]$	0.8285	0.8176	1.1165	2.763
Weight (w)	0.2999	0.2959	0.4041	1.000
Weight (w) in %	30.0	29.6	40.4	
Maximum of PCI	373,234	344,628	410,248	
Contributions	111,937	101,990	165,800	**379,727**

**Table 4:** Case-by-case entropy measures.

Exp. No.	Entropy SCA	Entropy CSA	Entropy ICA			Pi	Ln(pi + 1) − Ln(pi)	Yi = Pi*^(Ln(pi + 1) − Ln(pi))	1/Yi
1	−0.218	−0.199	−0.216			0.000	0.710	0.500	2.000
2	−0.224	−0.177	−0.220			0.693	0.693	0.480	2.081
3	−0.188	−0.167	−0.226			1.099	0.405	0.445	2.245
4	−0.197	−0.191	−0.173			1.386	0.288	0.399	2.507
5	−0.239	−0.221	−0.185			1.609	0.223	0.359	2.784
6	−0.237	−0.261	−0.273			1.792	0.182	0.327	3.061
7	−0.245	−0.263	−0.337			1.946	0.154	0.300	3.334
8	−0.249	−0.265	−0.170			2.079	0.134	0.278	3.601
9	−0.239	−0.252	−0.243			2.197	0.118	0.259	3.864
10	−0.253	−0.266	−0.156			2.303	0.105	0.243	4.122
** $En_{j}=-Y\sum_{i=1}^{n}Pr_{ij}log_{e}(Pr_{ij})$ **			$Div_{j}=\left|1-En_{j}\right|$ ** $+S_{q}$ **			**SUM of** **entropies**	**EW for** **SCA**	**EW for** **CSA**	**EW for ICA**
−0.436	−0.398	−0.432	0.603	0.562	0.271	1.436	0.420	0.392	0.189
−0.466	−0.369	−0.457	0.632	0.534	0.296	1.462	0.433	0.365	0.202
−0.423	−0.374	−0.508	0.589	0.539	0.346	1.475	0.400	0.366	0.235
−0.495	−0.478	−0.435	0.661	0.643	0.273	1.577	0.419	0.408	0.173
−0.666	−0.615	−0.515	0.832	0.779	0.354	1.966	0.423	0.397	0.180
−0.725	−0.800	−0.834	0.892	0.965	0.673	2.530	0.352	0.381	0.266
−0.816	−0.877	−1.124	0.982	1.042	0.963	2.987	0.329	0.349	0.322
−0.897	−0.954	−0.614	1.064	1.118	0.453	2.635	0.404	0.424	0.172
−0.925	−0.974	−0.938	1.091	1.139	0.776	3.006	0.363	0.379	0.258
−1.045	−1.097	−0.643	1.211	1.262	0.482	2.955	0.410	0.427	0.163

**Table 5:** Results of comparative methods.

Real Estate Appraisal		EWM		WEM 2 Adjustments		
Average	Weighted Average	WEM 1	WEM 2	Adjust. for SCA	Adjust. for CSA	Adjust. for ICA
244,636	252,905	379,727	369,012	156,649	134,976	77,387
235,268	247,722	379,727	370,275	161,446	125,845	82,985
201,346	203,442	379,727	371,464	149,166	126,000	96,298
260,370	247,643	379,727	367,991	156,428	140,458	71,104
298,169	303,986	379,727	368,555	158,021	136,666	73,867
273,770	296,469	379,727	372,170	131,550	131,466	109,153
260,609	294,941	379,727	375,192	122,711	120,200	132,281
353,553	354,730	379,727	367,450	150,677	146,294	70,478
281,554	300,682	379,727	371,952	135,439	130,585	105,928
376,037	372,055	379,727	371,952	152,944	147,217	66,888
278,531	287,458	379,727	370,601	147,503	133,971	88,637

## Data Availability

The data presented in this study are available on request from the corresponding author.
